# Immunogenicity of JN.1- and KP.2-Encoding mRNA COVID-19 Vaccines Against JN.1 Subvariants in Adult Participants

**DOI:** 10.1093/ofid/ofaf705

**Published:** 2025-11-19

**Authors:** Amparo L Figueroa, Bethany Girard, Darin K Edwards, Arshan Nasir, Kimball Johnson, Steven Hack, Xin Cao, Elizabeth de Windt, Veronica Urdaneta, Frances Priddy, Rituparna Das, David C Montefiori, Spyros Chalkias

**Affiliations:** Moderna, Inc., Cambridge, Massachusetts, USA; Moderna, Inc., Cambridge, Massachusetts, USA; Moderna, Inc., Cambridge, Massachusetts, USA; Moderna, Inc., Cambridge, Massachusetts, USA; CenExel, Decatur, Georgia, USA; Moderna, Inc., Cambridge, Massachusetts, USA; Moderna, Inc., Cambridge, Massachusetts, USA; Moderna, Inc., Cambridge, Massachusetts, USA; Moderna, Inc., Cambridge, Massachusetts, USA; Moderna, Inc., Cambridge, Massachusetts, USA; Moderna, Inc., Cambridge, Massachusetts, USA; Department of Surgery, Duke University Medical Center, Durham, North Carolina, USA; Moderna, Inc., Cambridge, Massachusetts, USA

**Keywords:** immunogenicity, JN.1 vaccine, KP.2 vaccine, mRNA-1273, SARS-CoV-2

## Abstract

In this ongoing, open-label, phase 3b/4 study, JN.1- and KP.2-encoding monovalent mRNA-1273 vaccines elicited robust neutralization of vaccine-matched variants (JN.1, KP.2) and cross-neutralized JN.1 subvariants circulating during the study (September 2024–November 2024; KP.3.1.1, XEC, LP.8.1) in vaccinated adults, showing reduced cross-neutralization against all subvariants tested. No safety concerns were identified.

Rapid spread and diversification of the SARS-CoV-2 Omicron lineage led to the emergence of the JN.1 variant, which became globally dominant by early 2024 [1]. Further diversification of JN.1 [2], led to authorization of the monovalent JN.1- and KP.2-encoding mRNA COVID-19 vaccines (2024–2025 formula) [3, 4]. Preclinical versions of these vaccines evaluated in naive (2-dose primary series) and previously immunized mice (booster) neutralized vaccine-matched variants and cross-neutralized JN.1 subvariants KP.2, KP.3, LA.2, and XEC (XEC in naive mice only) [5], supporting variant selection and updated vaccine approvals.

This study was conducted as part of an ongoing trial (NCT06585241) aiming to generate human serology data to assess immunogenicity and cross-neutralization of updated variant formulations of mRNA-1273 as new SARS-CoV-2 variants emerge.

Coinciding with this study period (September 9, 2024–November 11, 2024), newer JN.1 subvariants became dominant, including KP.3.1.1 and XEC, while LP.8.1 was emerging [6]. XEC, a recombinant of two JN.1 subvariants (KS.1.1 and KP.3.3) carried 2 additional mutations (T22N, F59S) compared with the previously dominant KP.3, and lacked S31del compared with KP.3.1.1 [7, 8]. LP.8.1, a KP.1.1.3 subvariant, contained 9 new spike mutations versus JN.1 and 6 versus KP.2 [7, 9, 10]. LP.8.1 started to increase in frequency in late November 2024 [10], became dominant in the United States by March 2025 [6], and is now the strain composition recommended for the 2025–2026 COVID-19 vaccine formulation in the United States [11]. While LP.8.1 seemed to be no more immune-evasive than XEC, it showed enhanced spike-hACE2 engagement, which likely facilitated its gradual and eventual replacement over XEC [7]. Both variants were classified as variants under monitoring by the World Health Organization [2] when the study was conducted, accounting for 68% of sequenced variants (LP.8.1: 60%; XEC: 8%) in the United States as of May 2025 [6].

Here, we evaluated the immunogenicity and cross-neutralization of the previously authorized 2024–2025 JN.1-encoding vaccine (mRNA-1273.167) and the KP.2-encoding vaccine (mRNA-1273.712) against vaccine-matched variants and SARS-CoV-2 variants circulating during the study period (KP.3.1.1, XEC, LP.8.1) [2, 6] in adults with previous COVID-19 mRNA vaccination; safety was also evaluated.

## METHODS

### Study Design and Participants

This is an ongoing, open-label, single-arm, phase 3b/4 study to evaluate the immunogenicity and safety of the 2024–2025 monovalent formulations of mRNA-1273 encoding the SARS-CoV-2 Omicron variants JN.1 (mRNA-1273.167) and KP.2 (mRNA-1273.712). Eligible participants were US adults (≥18 years) with ≥3 previous mRNA COVID-19 vaccinations (at least 2-dose primary series of mRNA original monovalent and an XBB.1.5-encoding mRNA COVID-19 vaccine received between September 2023 and August 2024). Adults with history of SARS-CoV-2 infection within 3 months before enrollment, as assessed by medical history, were excluded (Supplementary Appendix). Participants received a 0.5-mL dose of a single intramuscular injection (50 µg) of either mRNA-1273.167 (Subprotocol 1) or mRNA-1273.712 (Subprotocol 2) and were followed for 1 month. The study is being conducted in accordance with the Declaration of Helsinki and Council for International Organizations of Medical Sciences international ethical guidelines, International Council for Harmonisation good clinical practice guidelines, and applicable laws and regulations.

### Patient Consent Statement

The protocol, informed consent form, and other relevant documents were reviewed and approved by the institutional review board/independent ethics committee (Advarra, Columbia, MD, USA) before study initiation. Written informed consent was obtained prior to study enrollment.

### Study Vaccines

mRNA-1273.167 (Spikevax, Moderna, Inc.) contains 50 µg of mRNA encoding the full-length SARS-CoV-2 spike protein of the JN.1 variant with 2 proline residue substitutions to stabilize the spike protein into a prefusion conformation. mRNA-1273.712 contains 50 µg of mRNA encoding the prefusion-stabilized spike protein of the KP.2 variant.

### Immunogenicity, SARS-CoV-2, and Safety Assessments

Sera for neutralizing antibody (nAb) response assessments were collected before vaccine administration (Day 1) and 4 weeks after immunization (Day 29). The nAb titers were quantified using a lentivirus-based SARS-CoV-2 pseudovirus neutralization assay (Duke University) [12], updated for JN.1, KP.2, KP.3.1.1, XEC, and LP.8.1 (Supplementary Appendix). Positive baseline SARS-CoV-2 status was determined by virologic (reverse transcription polymerase chain reaction [RT-PCR]) and/or serologic (antinucleocapsid binding antibody) evidence of SARS-CoV-2 infection on or before Day 1; negative status was determined by a negative RT-PCR test and a negative serology test on or before Day 1 (Supplementary Appendix). Safety assessments included adverse events (AEs) leading to study withdrawal, serious AEs (SAEs), and AEs of special interest (AESIs) from Day 1 through the end of the study (Day 29).

### Statistical Analysis

Immunogenicity was evaluated in the per-protocol immunogenicity set (PPIS), comprising all participants who received a planned study vaccine, had a negative RT-PCR test at Days 1 and 29, and had no major protocol deviations impacting the key data. The primary endpoint was geometric mean titer (GMT) and geometric mean fold rise (GMFR) of nAbs at Day 29 relative to baseline, with corresponding 95% confidence intervals. Safety was assessed in the safety set (all enrolled participants who received study intervention). No statistical hypothesis testing was performed; primary immunogenicity results are descriptive.

## RESULTS

Overall, 50 participants were independently enrolled in each subprotocol and received mRNA-1273.167 (Subprotocol 1; median age, 63 years; 62% female; 86% Black/African American) or mRNA-1273.712 (Subprotocol 2; median age, 54 years; 66% female; 70% Black/African American). Median time on study was 30 days (mRNA-1273.167) and 29 days (mRNA-1273.712). Overall, 76–80% of participants received 3 prior vaccinations, 12–20% received 4 prior vaccinations, and 4–8% received ≥5 prior vaccinations across subprotocols (median time [interquartile range, IQR] since last dose: mRNA-1273.167, 310 days [303–316]; mRNA-1273.712, 330 days [277–338]). No participants had a medical history of SARS-CoV-2 infection within the past 3 months. In Subprotocol 1, 39 (78.0%) participants tested positive for SARS-CoV-2 by serology at baseline; the PPIS included 48 (96.0%) participants (two were excluded due to a missing or positive SARS-CoV-2 RT-PCR result on Day 29). In Subprotocol 2, 43 (86.0%) participants tested positive for SARS-CoV-2 by serology at baseline; the PPIS included 49 (98.0%) participants (one was excluded due to a positive or missing SARS-CoV-2 RT-PCR result on Day 29).

**Figure 1.**
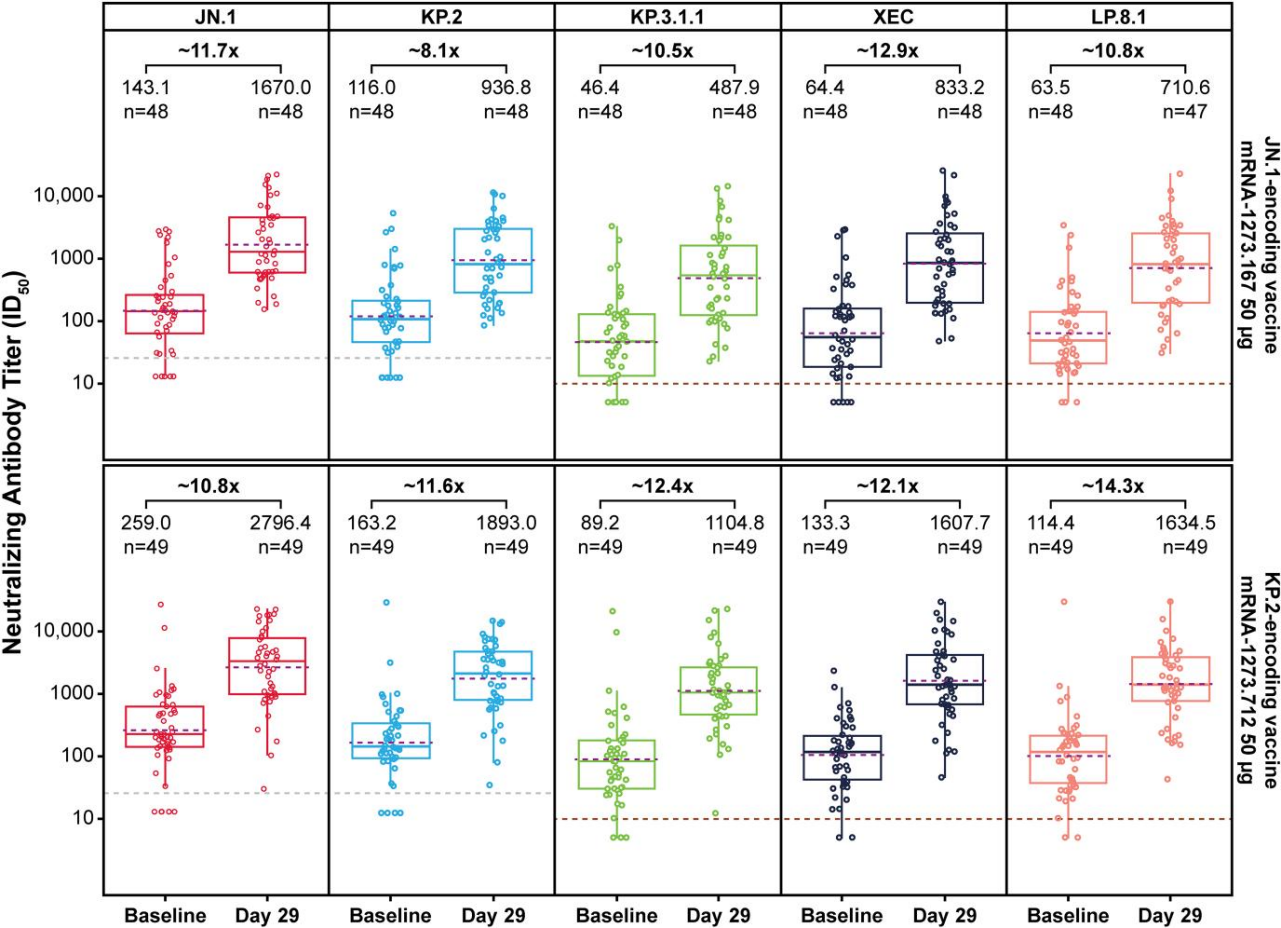
Neutralizing antibody responses (GMT and GMFR) elicited by mRNA-1273.167 (JN.1-encoding vaccine) and mRNA-1273.712 (KP.2-encoding vaccine) against the vaccine-matched variants (JN.1, KP.2) and newly emerged subvariants (KP.3.1.1, XEC, LP.8.1), per-protocol immunogenicity set. The GMT (Day 1 and Day 29) with the corresponding GMFR (Day 29 relative to Day 1) are shown. The PPIS included participants who received the planned study intervention, had negative RT-PCR tests at baseline (Day 1) and Day 29, and had no major protocol deviations that impacted the key data. Participants were excluded from the PPIS if they had a missing or positive SARS-CoV-2 RT-PCR test result on either Day 1 or Day 29. The boundary of boxes represents the 25th (bottom) and 75th (top) percentiles of the GMT. The solid line inside the box represents the median (50th percentile) of the GMT. The dashed line inside the box represents the GMT. Whiskers (vertical lines) represent the lowest and highest data points within 1.5 of the interquartile range. The LLOQ is presented using a lighter dashed line. The LOD is presented using a darker dashed line. Abbreviations: GMFR, geometric mean fold rise; GMT, geometric mean titer; ID_50_, 50% inhibitory dilution; LLOQ, lower limit of quantification; LOD, limit of detection; PPIS, per-protocol immunogenicity set; RT-PCR, reverse transcription polymerase chain reaction.

**Figure 2.**
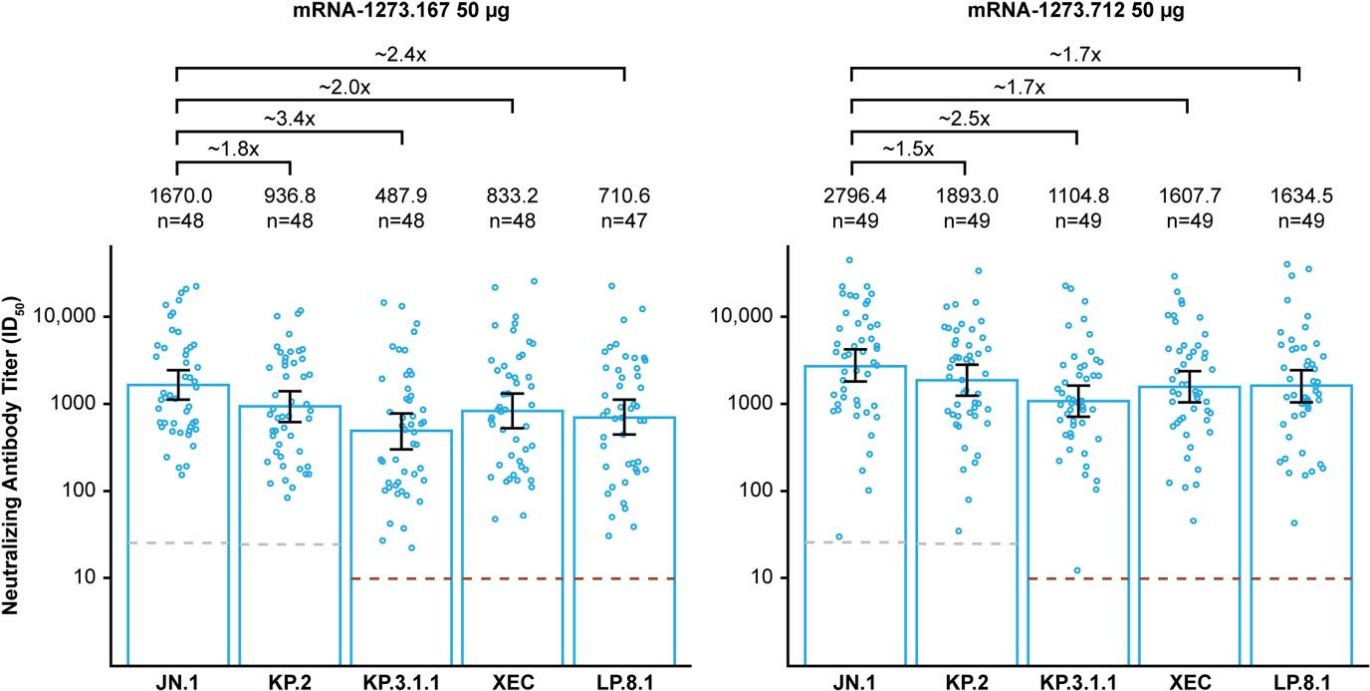
Geometric mean fold-drop in variant-specific neutralizing antibody responses at Day 29 after mRNA-1273.167 (JN.1-encoding vaccine) and mRNA-1273.712 (KP.2-encoding vaccine), per-protocol immunogenicity set. The LLOQ is presented using a lighter dashed line. The LOD is presented using a darker dashed line. Annotated fold change indicates fold-drop compared with the reference variant JN.1. Abbreviations: ID_50_, 50% inhibitory dilution; LLOQ, lower limit of quantification; LOD, limit of detection.

Both JN.1-encoding (mRNA-1273.167) and KP.2-encoding (mRNA-1273.712) vaccines robustly increased nAb responses at Day 29 relative to baseline against matched variants (GMFR, 11.6–11.7) and cross-neutralized JN.1-lineage subvariants not matched to the vaccine (GMFR, mRNA-1273.167: KP.2, 8.1; KP.3.1.1, 10.5; XEC, 12.9; LP.8.1, 10.8; GMFR, mRNA-1273.712: JN.1, 10.8; KP.3.1.1, 12.4; XEC, 12.1; LP.8.1, 14.3; Figure 1). The highest titers were measured against JN.1 for both vaccines (GMT: mRNA-1273.167, 1670.0; mRNA-1273.712, 2796.4). Relative to the JN.1 reference, a reduction in cross-neutralization was observed against JN.1-lineage subvariants (KP.3.1.1, XEC, LP.8.1) for both vaccines, with the greatest reduction measured against KP.3.1.1 (Figure 2). Higher nAb responses were elicited by mRNA-1273.712 than mRNA-1273.167 against all tested variants (Figure 2).

No SAEs, deaths, or AEs leading to study withdrawal were reported throughout the study. One AESI (pulmonary embolism) was reported in the study, which was considered unrelated to vaccination by the investigator. At 23 days postvaccination in the JN.1 cohort, a participant in their 50s with hypothyroidism presented to the emergency department with chest pain/diaphoresis; a small pulmonary embolism was found, and the participant was treated with anticoagulants and then discharged on oral anticoagulants.

## DISCUSSION

In this phase 3b/4 study evaluating adults with previous COVID-19 mRNA vaccination, monovalent JN.1- and KP.2-encoding mRNA-1273 vaccines (mRNA-1273.167 and mRNA-1273.712) induced robust nAb responses against matched variants and cross-neutralized newer JN.1 subvariants (KP.3.1.1, XEC, LP.8.1). Cross-neutralization was reduced across all subvariants versus JN.1. No safety concerns were identified over the 1-month follow-up period. While both vaccines induced cross-neutralization consistent with the expected potent cross-reactivity of the JN.1- and KP.2-encoding vaccines against emerging JN.1-lineage subvariants [5, 9], decreased cross-neutralization measured against KP.3.1.1, XEC, and LP.8.1 for both vaccines suggested that newer variants developed some immune escape from responses induced by the previously approved 2024–2025 COVID-19 vaccine compositions. At Day 29, the KP.2-encoding vaccine (mRNA-1273.712) induced higher nAb responses than the JN.1-encoding vaccine (mRNA-1273.167) against all variants tested, suggesting an advantage in preventing escape. However, higher baseline titers in the KP.2 cohort may have influenced higher nAb titers and fold-rises at Day 29, although greater GMFRs for KP.2, KP.3.1.1, and LP.8.1 were observed in the KP.2 cohort despite higher baseline titers. The highest reduction in cross-neutralization was observed for KP.3.1.1, consistent with data reporting increased resistance to serum neutralization of KP.3.1.1 versus XEC in KP.2 vaccine recipients [13]. The drop in cross-neutralization versus the JN.1 reference ranged between 1.5 and 3.4 after the JN.1- and KP.2-encoding vaccines, which was lower than that reported against JN.1 (∼5.8-fold reduction) after booster vaccination with the XBB.1.5-encoding mRNA-1273 vaccine authorized for the 2023–2024 season [12]. These data are consistent with the reported cross-neutralization of KP.2 monovalent boosters against JN.1 subvariants, including KP.3.1.1 and XEC, and with observations of reduced neutralization against these subvariants versus JN.1 or KP.2 [14, 15]. Similar findings were reported for the JN.1-encoding mRNA booster among healthcare workers, with the highest neutralization measured against JN.1, and 1.9- and 2.9-fold lower titers measured against KP.3.1.1 and XEC, respectively [16].

The updated 2025–2026 mRNA-1273 COVID-19 vaccine formulation in the United States now targets the JN.1 sublineage LP.8.1 [17]. Vaccine efficiency (compared to placebo) and immunogenicity of LP.8.1-encoding mRNA-1273 will be evaluated in adults 50 to 64 years at no risk of severe COVID-19; in addition, immunogenicity of LP.8.1-encoding mRNA-1273 will be evaluated in individuals with risk factors for severe COVID-19 outcomes, including adolescents and adults (≥12 years; NCT06585241) and pediatric (≥6 months to <12 years) populations. In light of the continued SARS-CoV-2 evolution, these data underscore the importance of continuously assessing the immunogenicity and cross-neutralization of updated variant formulations of COVID-19 vaccines.

Study limitations include small sample size and lack of durability assessments of nAb responses. Participant enrollment in the JN.1 and KP.2 vaccine arms was nonrandomized, and comparisons should be made with caution.

In conclusion, the JN.1- and KP.2-encoding monovalent mRNA-1273 vaccines elicited robust nAb responses against matched variants and cross-neutralized currently circulating JN.1 subvariants (KP.3.1.1, XEC, LP.8.1), with reduced cross-neutralization observed against all subvariants tested. No safety concerns were identified.

## Supplementary Material

ofaf705_Supplementary_Data

## Supplementary Data

Supplementary materials are available at *Open Forum Infectious Diseases* online. Consisting of data provided by the authors to benefit the reader, the posted materials are not copyedited and are the sole responsibility of the authors, so questions or comments should be addressed to the corresponding author.
